# Deconvoluting complex correlates of COVID-19 severity with a multi-omic pandemic tracking strategy

**DOI:** 10.1038/s41467-022-32397-8

**Published:** 2022-08-30

**Authors:** Victoria N. Parikh, Alexander G. Ioannidis, David Jimenez-Morales, John E. Gorzynski, Hannah N. De Jong, Xiran Liu, Jonasel Roque, Victoria P. Cepeda-Espinoza, Kazutoyo Osoegawa, Chris Hughes, Shirley C. Sutton, Nathan Youlton, Ruchi Joshi, David Amar, Yosuke Tanigawa, Douglas Russo, Justin Wong, Jessie T. Lauzon, Jacob Edelson, Daniel Mas Montserrat, Yongchan Kwon, Simone Rubinacci, Olivier Delaneau, Lorenzo Cappello, Jaehee Kim, Massa J. Shoura, Archana N. Raja, Nathaniel Watson, Nathan Hammond, Elizabeth Spiteri, Kalyan C. Mallempati, Gonzalo Montero-Martín, Jeffrey Christle, Jennifer Kim, Anna Kirillova, Kinya Seo, Yong Huang, Chunli Zhao, Sonia Moreno-Grau, Steven G. Hershman, Karen P. Dalton, Jimmy Zhen, Jack Kamm, Karan D. Bhatt, Alina Isakova, Maurizio Morri, Thanmayi Ranganath, Catherine A. Blish, Angela J. Rogers, Kari Nadeau, Samuel Yang, Andra Blomkalns, Ruth O’Hara, Norma F. Neff, Christopher DeBoever, Sándor Szalma, Matthew T. Wheeler, Christian M. Gates, Kyle Farh, Gary P. Schroth, Phil Febbo, Francis deSouza, Omar E. Cornejo, Marcelo Fernandez-Vina, Amy Kistler, Julia A. Palacios, Benjamin A. Pinsky, Carlos D. Bustamante, Manuel A. Rivas, Euan A. Ashley

**Affiliations:** 1 Department of Medicine, Stanford University School of Medicine, Stanford, CA USA; 2 Department of Biomedical Data Science, Stanford University, Stanford, CA USA; 3 Institute for Computational and Mathematical Engineering, Stanford University, Stanford, CA USA; 4 Department of Genetics, Stanford University School of Medicine, Stanford, CA USA; 5 Histocompatibility & Immunogenetics Laboratory, Stanford Blood Center, Stanford Health Care, Stanford, USA; 6 Department of Statistics, Stanford University, Stanford, CA USA; 7 Department of Aeronautics and Astronautics, Stanford University, Stanford, CA USA; 8 Department of Computational Biology and Swiss Institute of Bioinformatics, University of Lausanne, Lausanne, Switzerland; 9 Department of Computational Biology, Cornell University, Ithaca, NY USA; 10 Department of Pathology, Stanford University School of Medicine, Stanford, CA USA; 11 Medical Scientist Training Program, University of Pittsburgh and Carnegie Mellon University, Pittsburgh, PA USA; 12 Chan Zuckerburg Biohub, San Francisco, CA USA; 13 Department of Bioengineering, Stanford University, Stanford, CA USA; 14 Sean N. Parker Center for Allergy and Asthma Research, Stanford University School of Medicine, Stanford, CA USA; 15 Department of Emergency Medicine, Stanford University School of Medicine, Stanford, CA USA; 16 Department of Psychiatry and Behavioral Sciences, Stanford University School of Medicine, Stanford, CA USA; 17 Takeda Development Center, Americas, Inc, San Diego, CA USA; 18 Illumina, Inc, San Diego, CA USA; 19 School of Biological Sciences, Washington State University, Pullman, WA USA

**Keywords:** Genetics, Sequencing, Viral infection

## Abstract

The SARS-CoV-2 pandemic has differentially impacted populations across race and ethnicity. A multi-omic approach represents a powerful tool to examine risk across multi-ancestry genomes. We leverage a pandemic tracking strategy in which we sequence viral and host genomes and transcriptomes from nasopharyngeal swabs of 1049 individuals (736 SARS-CoV-2 positive and 313 SARS-CoV-2 negative) and integrate them with digital phenotypes from electronic health records from a diverse catchment area in Northern California. Genome-wide association disaggregated by admixture mapping reveals novel COVID-19-severity-associated regions containing previously reported markers of neurologic, pulmonary and viral disease susceptibility. Phylodynamic tracking of consensus viral genomes reveals no association with disease severity or inferred ancestry. Summary data from multiomic investigation reveals metagenomic and HLA associations with severe COVID-19. The wealth of data available from residual nasopharyngeal swabs in combination with clinical data abstracted automatically at scale highlights a powerful strategy for pandemic tracking, and reveals distinct epidemiologic, genetic, and biological associations for those at the highest risk.

## Abstract (web summary)

There is a genetic component to the risk of severe COVID-19, but the genetic effects are difficult to separate from social constructs that covary with genetic ancestry. To address this, the authors identify determinants of COVID-19 severity using admixture mapping, viral phylodynamics, and host immune and metagenomic sequencing.

## Introduction

Two central questions from the COVID-19 pandemic remain unresolved: who is at risk of severe disease, and why? Genome-wide-association-studies (GWAS) from the COVID-19 Host Genetics Initiative and others have identified up to 13 genetic loci associated with COVID-19 infection, hospitalization and critical illness^1–3^. Among these loci are the ABO blood type locus, a variant found at high frequency in Pacific Islander populations^4^, and a chromosome 3 haplotype that is shared with the Neanderthal genome and overrepresented in individuals of European ancestry, all suggesting that genetic ancestry can play a role, albeit small, in susceptibility and severity in SARS-CoV-2 infection^5^. At the same time, epidemiologic studies have shown that comorbidities, sex, and race/ethnicity are strongly associated with infection prevalence and disease severity^6–9^. For example, several groups have reported higher incidence of COVID-19 and higher disease severity among Hispanic/Latino and African American racial and ethnic groups^6,8^. Because the social constructs of race and ethnicity can covary with overall genetic ancestry (e.g., as examined in the COVID-19 Host Genomics Initiative^3,10^) and because such overall ancestry lacks the complexity of local genomic context, such associations may confound the study of COVID-19 host genetic susceptibility by inappropriately associating markers of genetic ancestry with disease severity.

To eliminate this confounding, we used genetic ancestry inference along the genome: After controlling for individual genetic ancestry proportions, we use local ancestry inference to label each segment of an individual’s genome by its ancestral origin and then identify associations of each of these segments with disease severity (as compared across a composite genome from the same ancestry). This eliminates socioeconomic and environmental confounders because independent assortment of parental chromosomes and recombination within them shuffle these ancestral haplotypes, resulting in random differences even between siblings in the same household with ostensibly the same socioeconomic pressures. In addition to this analysis, we examined viral variants, host immunity (e.g., HLA typing), and the microbiome as covariates of majority ancestry to identify important potential contributors to disease severity.

## Results

**Fig. 1:**
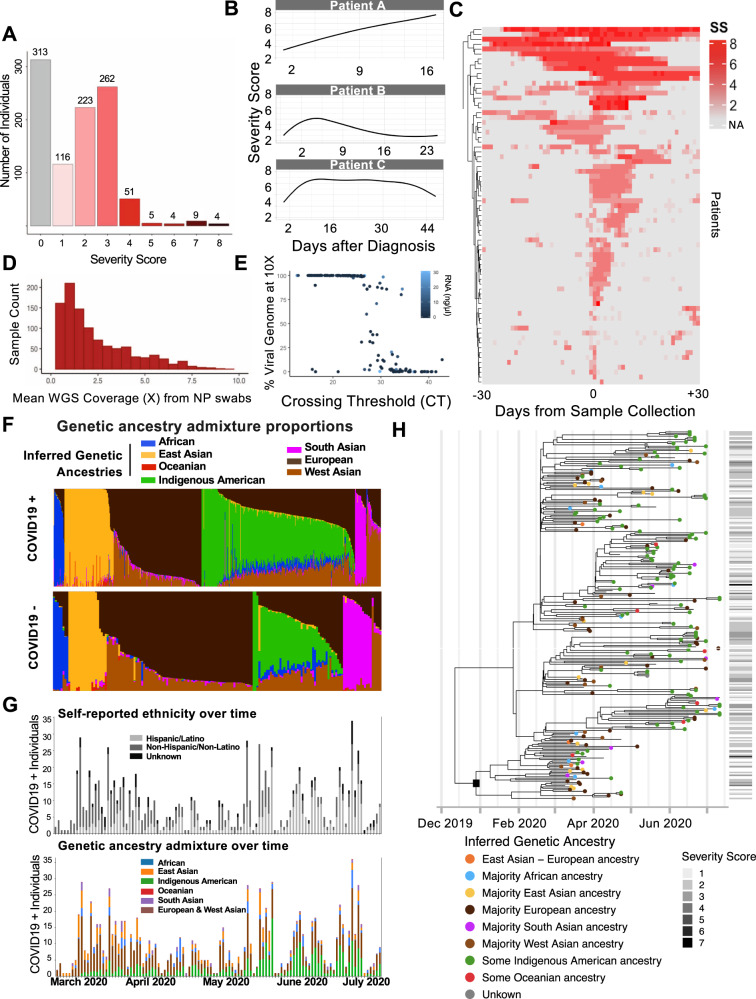
SARS-CoV-2 pandemic tracking from residual NP swabs and abstracted EHR data combined with genetic ancestry inference allows identification of high risk populations and examination of its interaction with viral phylogeny and disease severity. **A** We collected samples from 736 SARS-CoV2 positive and 313 negative patients between Mar-Aug 2020 with clinical severity scores ranging from 1 (ambulatory) to 8 (death). **B** Examples of individual patient trajectories in COVID-19 severity score as abstracted from the electronic healthcare record. **C** Severity scores abstracted directly from the electronic health record daily for thirty days before and after the positive NP swab test on all included patients with severity score ≥ 4 (hospitalized, needs oxygen) demonstrates significant variability in patient course. **D** Whole genome sequencing from DNA isolated from 150 ul of NP swab VTM yielded sequence on >95% of samples with mean of means coverage 2.6X. **E** RNA sequencing using shotgun sequencing recovered consensus SARS-CoV-2 sequence on the majority of NP swabs with a clinical PCR CT value <30. ARTIC primer enrichment increased this yield (Supplementary Fig. 1D). **F** Genetic ancestry admixture of individuals with positive versus negative COVID-19 tests in the present study. Individuals with Indigenous American ancestry are overrepresented in cases, whereas controls show more European and South Asian genetic ancestry. **G** Self-reported (top) and genetic ancestry (bottom) of enrolled COVID-19 + individuals over time reveals disproportionate representation of Hispanic/Latino ethnicity and Indigenous American ancestries during summer pandemic wave, whereas the first wave is seen to have predominantly affected non-Hispanic individuals and individuals of European genetic ancestry. **H** Phylogenetic reconstruction of SARS-CoV-2 sequences. Tip colors correspond to the inferred genetic ancestry of the infected hosts, whose consensus SARS-CoV-2 sequences were isolated and used for inferring the viral phylogeny. Horizontal lines to the right of the phylogeny indicate host severity scores corresponding to the tips of the phylogeny. Severity score codes are displayed in Supplementary Table 1.

Residual viral transport media (VTM) from SARS-CoV-2 clinical diagnostic tests were prospectively collected from March 2020 to July 2020 from Stanford Health Care in northern California, USA. Swabs were selected approximately consecutively from SARS-CoV-2 positive and negative individuals and linked to structured clinical information from the electronic health record. In total, 1327 NP swab residuals were collected from 1049 individuals (736 positive and 313 negative, Fig. 1A and S2). For digital phenotype abstraction, we developed a method to generate a COVID-19 clinical severity score automatically from the electronic health record based on the ordinal scale proposed by the World Health Organization (Supplementary Table 1 and Supplementary Fig. 2). Clinical data were obtained through the STAnford Research Repository (STARR), specifically the STRIDE data management system, which is populated from patients’ clinical and biospecimen data^11^. Severity scores were calculated on the date of sample collection and daily for one month before and indefinitely after (Fig. 1B, C, S2, 4). Host whole genome sequencing was aligned and called using methods for low pass data^12–14^ (mean of mean coverages: 2.56X ± 2.50X(SD), Fig. 1D), followed by phasing and imputation with GLIMPSE (Supplementary Fig. 1A, B)^15^. Shotgun RNAseq of initial samples yielded high coverage of much of the viral genome for samples with clinical test CT values <35 regardless of RNA yield, which improved with primer-based capture (Fig. 1E and S1D).

We used genetic ancestry inference to identify subpopulations highly impacted by the COVID-19 pandemic. Self-reported race and ethnicity re-demonstrates prior reports of over-representation of minority race and ethnic populations within the US amongst COVID-19+ patients (Supplementary Fig. 3A, B). Based on genome-wide ancestry inference, a higher proportion of individuals of Indigenous American genetic ancestry exists in COVID-19 cases as compared to negative controls (Fig. 1F, 44% vs. 26%, χ^2^ = 99, *p* < 1e−10 for individuals having Indigenous American genetic ancestry >10%, characteristic of Hispanic populations). This association persists even when adjusted for age, sex, and BMI (*p* = 1.95*10^−3). The association with the proportion of inferred Indigenous American ancestry, after again adjusting for age, sex, and BMI, is even more significant (4.64*10^−4), suggesting a connection between this ancestry and socioeconomic links to exposure factors. Indeed, evaluation of temporal trends of genetic ancestry in case positivity reveals early predominance of European ancestry followed by a significant increase in Indigenous American ancestry after May 2020. These findings are recapitulated by self-reported ethnicity, as the majority of COVID-19 cases between May and July 2020 self-identified as Hispanic/Latino in the medical record, an ethnicity often associated with admixed Indigenous American and European genetic ancestry (Fig. 1G).

We investigated the role of viral phylogenetics in COVID-19 severity and its potential interaction with individual ancestry and disease severity. Consensus viral genomes (10X coverage at >99% of the genome) were recovered for 255 samples from unique, unrelated individuals. The estimated time to the most recent common ancestor of observed samples is December 11, 2019 with a 95% Bayesian CI of (2019-10-27, 2020-01-12). However, the phylogenetic reconstruction (Fig. 1H) reveals an early introduction in the area between late December 2019 and early January of 2020 with several independent introductions later in February 2020. While around 37% of the infected individuals in the sample have Indigenous American ancestry, there is no evidence of exclusive transmission amongst individuals of this ancestry. Other majority-vote genetic ancestries are also not associated with particular clades (*p* > 0.05^14^), though a single clade from early in the pandemic had fewer Hispanic individuals (lineage subtending the clade is marked with a square), consistent with the first wave prior to May 2020, in which European genetic ancestry individuals were enriched. We also tested the hypothesis of association between viral lineages and disease severity. No association at a significance level of 0.05 was found between specific clades and severity score at the time of NP swab in this early stage of the pandemic.

**Fig. 2:**
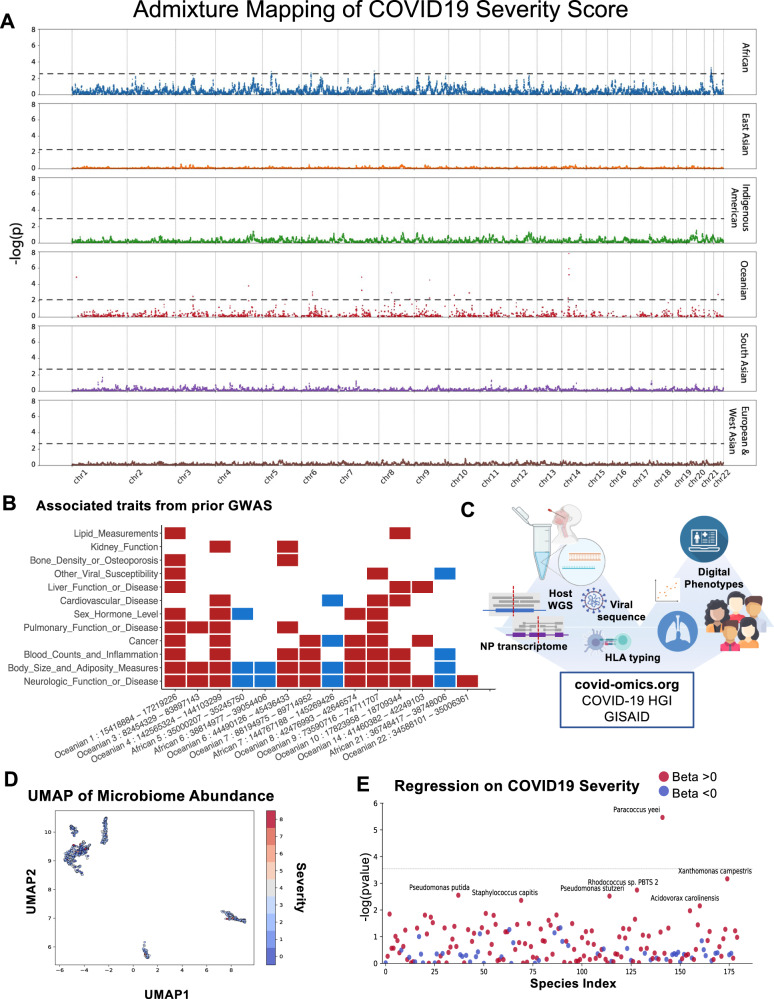
COVID-19 severity is associated with local-ancestry-specific risk loci via admixture mapping, and is also correlated with metagenomic features of the NP transcriptome. **A** Ancestry-specific risk loci found in African and Oceanian ancestries, respectively after correcting for overall genetic ancestry proportion, BMI, sex, and age. Each colored dot represents a window of the genome. Black lines represent ancestry-specific thresholds determined by the method of Shriner et al.^19^ Thresholds determined by running one thousand association tests on random permutations of case-control labels are displayed in Figure S5. **B** Traits associated with genomic regions statistically enriched for disease severity in the GWAS catalog. For additional information including a full list of previously reported SNPs and neighboring genes, see Supplementary Data 1. All summary statistics are available at covid-omics.org. **C** Schematic of multiomic pandemic tracking strategy. Created with BioRender.com. **D** Uniform manifold approximation and projection (UMAP) of patient Nasal Microbiome abundances colored by patient COVID-19 severity score. (**E**) Regression of species-specific abundance against continuous disease severity, corrected for age, sex and BMI, identified *P. yeei* abundance in the nasopharyngeal microbiome as associated with high severity COVID-19 infections (Bonferroni adjusted *p* = 7e−04 (two-sided)).

After adjusting for age, sex and BMI (known correlates of disease severity^16–18^) together with overall genetic ancestry proportion, we assessed association of genetic ancestry at a given genomic position with the COVID-19 severity score as an ordinal outcome (admixture mapping). Because our captured case population was enriched for non-European ancestry groups, we were able to perform admixture mapping for six ancestries (African, Native American, Oceanian, South Asian, East Asian, and European/West Asian). This analysis revealed loci in chromosomal regions of African and Oceanic ancestry that met genome-wide significance (threshold determined as previously described by Shriner et al.)^19^ (Fig. 2A). SNVs in many of these regions have been previously associated with neurologic signs, and body size/adiposity in prior GWAS, as well as pulmonary traits, viral susceptibility, and hematologic characteristics (Fig. 2B, Supplementary Data 1). It is important to note that the absence of prior associations for GWAS severity (e.g., on chromosome 3) in these disaggregated samples may be indicative of reduced power to detect these associations based on the particular admixed populations of this cohort. As a large proportion of these COVID + patients did not carry majority European genomic ancestry, we may be under-powered to replicate such associations found in majority European ancestry populations prior. We also note that admixed East Asian ancestry was underrepresented in our COVID+ cohort, meaning that for stretches of the genome associated with East Asian ancestry this analysis was underpowered to identify smaller effect sizes. Critically, in fact *because* we perform admixture analysis, these results *cannot and do not imply that genetic risk associates with overall genetic ancestry*. Rather, the deconvolution made possible by ancestry admixture analysis can unmask novel biology.

We next established a web portal of summary statistics for COVID-19 severity versus host genotype by genetic ancestry, host HLA type, and metagenomic alignments (https://covid-omics.org/results). We also contributed host genetic summary data and viral consensus sequences to the COVID-19 Host Genetics Initiative^3^ and GISAID^20^, respectively (Fig. 2C). Using this resource, we explored the potential contribution of the nasopharyngeal microbiome and HLA-type as biological determinants of COVID-19 severity. A UMAP plot of microbiome species abundance shows clustering largely independent of severity (Fig. 2D). However, a regression of species abundance against COVID-19 severity (controlling for age, sex and BMI) revealed enrichment of *Paracoccus yeei* sequence in high severity cases. This is a bacterium that causes opportunistic infections in critically ill^21^, organ transplant^22^, and dialysis patients^23,24^, indicating an association with immune compromise and severe illness (Fig. 2E*p* = 3.58e−06 after Bonferroni correction). The HLA-B*07:02 allele (common prototype allele for the serotype B7) was associated with elevated risk of high severity score (OR 2.7 [1.4, 5.1], *p* = 2.9e−03), whereas the HLA-C*15:02 allele (common prototype allele for the serotype Cw15) was associated with risk reduction (OR 0.12 [0.02, 0.82], *p* = 1.41e−02) (Supplementary Fig. 3D). HLA-B*07:02 presents epitopes from the SARS-CoV-2 *N* gene and *Orf1ab*^25^, and the HLA-C*15:02 allele contains two distinctive amino acid substitutions at residues 113 and 116 located within the peptide binding groove. HLA-C*15:02 was associated with milder disease in the first SARS epidemic^26^, and is predicted to bind a SARS-CoV-2 Spike protein epitope^27^.

## Discussion

These results represent a substantial effort to assemble host and viral genomic, transcriptomic and digital clinical data from a diverse cross-section of the racial and ethnic groups affected by the COVID-19 pandemic. We show that ancestry inference can be used to track changes in the affected population in real-time, demonstrating that Hispanic/Latino groups (associated with Indigenous American genetic ancestry) were disproportionately affected during a second pandemic wave. This is consistent with the model that this second wave was driven not by introduction from travelers (likely the source of the first wave), but by economic pressure on essential service workers to leave their homes and family units, enabling viral spread^28^. Phylodynamic overlay on this at-risk population further supports this conclusion, demonstrating that viral clades did not differentially affect ancestral groups, nor did they confer differential disease severity during the six months of prospective enrollment. Thus, the impact of introduction of viral variants on community spread was likely less than that of exposure related to essential services work.

In addition to the use of ancestry inference to track the impact of the pandemic on ancestral populations, genomic regions associated with COVID-19 severity in the context of local African and Oceanian ancestries highlight potentially novel pathobiology: Nearby SNPs and genes have previously been associated in particular with other viral susceptibility, Alzheimer’s disease pathology, body size and adiposity measurements and pulmonary function and disease^29^. Admixture mapping and local ancestry disaggregation were necessary to reveal these markers, which would likely otherwise be masked by social and economic determinants of severity that disproportionately affect these populations.

Due in large part to health inequities, the populations at highest risk of severe outcomes are not proportionally represented in existing datasets. As such, the development of a real-time data collection strategy from clinical swab residuals was critical to assessing the relevance of ancestry-specific genetic variation^30^. We present resources combining summary host and viral genomic, metagenomic and transcriptomic data with digital phenotype abstraction from extant EHR data to help deconvolve genetic environmental and social factors while tracking spread across the community. This system can be applied in real-time to model individual and population trajectories in the face of future emerging global infectious disease (https://covid-omics.org/results). In addition, our work serves to illuminate COVID-19 disease biology that might otherwise be missed due to the confounding of social and economic factors that are critically associated with race and ethnicity in diverse populations.

## Methods

### Sample collection and diagnostics

This work complies with all relevant ethical regulations and was performed under protocol IRB-55580, which was approved by the Stanford University School of Medicine IRB; its most recent approval was 5/6/2021. Residual VTM from SARS-CoV-2 positive nasopharyngeal swabs collected during clinical assessment of asymptomatic and symptomatic patients at Stanford Healthcare were used in accordance with the Stanford School of Medicine Institutional Review Board. Participants were not compensated. Since samples were residual and not linked to identifiable medical records, our IRB classified this study as low risk and informed consent was waived. RT-qPCR targeting the *envelope* gene or ORF1ab was used to detect infection. Positive samples were defined as those crossing threshold (CT) of 40 cycles or less on the RT-qPCR or positive Transcription-Mediated Amplification (TMA) diagnostic tests used at Stanford Health Care clinical laboratory^31^. Where multiple samples were collected from the same individual, the COVID-19+ sample taken at the time of highest severity score was used for low pass WGS. Negative controls were confirmed to have no positive COVID-19 nasal swab tests in our system.

### EHR data abstraction and severity score development

A critical task is to determine for every sampled patient the disease severity from the Electronic Health Records (EHR). To accomplish this task, we used as the “COVID-19 Clinical Severity Scale” an adapted version of the “Ordinal Scale for Clinical Improvement” proposed by the World Health Organization in the COVID-19 Therapeutic Trial Synopsis (Draft February 18, 2020, Supplementary Table 1). This scale categorized the COVID-19 severity according to the level of care and oxygen support. Scores 1 to 2 include patients not requiring supplemental oxygen support or hospitalization. However, the WHO definition of these scores were modified due to their vague scope. Thus, score “1”, originally described as “no limitation of activities”, was modified to “asymptomatic patient”, and score “2”, from “limitation of activities” to “symptomatic patient” (symptoms were extracted and curated from EHR billed diagnoses). Scores 3 to 4 include patients hospitalized, with score 4 assigned only to those requiring non-invasive supplemental oxygen (oxygen mask). Scores 5 to 7 are defined as “severe disease” based on level of oxygen support. Thus, “score 5” is for patients requiring high flow oxygen and “score 6” mechanical ventilation. “Score 7” includes critically ill patients requiring, in addition to ventilation, the administration of specific medications (pressors), dialysis, or extracorporeal membrane ventilation (ECMO). A custom algorithm was written that abstracted digital phenotypes from each chart (see Supplementary Table 1, “EHR annotations”). First, SARS-CoV-2 positive status was confirmed based on clinical test reports abstracted from the EHR. SARS-CoV-2 negative patients were assigned a score of zero. For SARS-CoV-2 positive patients, starting with the highest score (8, death) and working down, if criteria were met, the individual was assigned that score. If no clinical notes were available for data abstraction, then a severity score was not assigned and these individuals were not included in severity score based analyses. We calculated the score for any given date and assigned the maximum value according to the EHR annotations defined for every score (Supplementary Table 1). For example, patients with annotations for both ventilation and the administration of pressors received a score “7” for that day. Clinical data were obtained through the STAnford Research Repository (STARR), a Stanford Medicine’s approved resource for working with clinical data for research purposes extracted from the Epic database management system used by the Stanford hospitals. Specifically, we queried the STRIDE data management system, which is populated from patients’ observational clinical, research, and biospecimen data^11^. A summary of characteristics of patients included in each analysis described below is available in Supplementary Data 2.

### Nucleic acid extraction

Host genomic DNA was extracted from 200ul of VTM inoculated with nasopharyngeal swabs. Using a modified Qiagen DNEASY blood and tissue kit protocol and quantified using fluorometric readings (Protocols.io 10.17504/protocols.io.bi8xkhxn). Total RNA was extracted from 200ul of VTM using a modified Ambion mirVana mRNA kit protocol (Protocols.io 10.17504/protocols.io.bi8ykhxw) or Zymo Research Quick-Viral RNA extraction kits (R1041) and quantified using fluorometric readings.

### Host gDNA library preparation and sequencing

Using 1-10 ng of host gDNA, the Illumina Nextera Flex library preparation was performed according to manufacturer’s protocol (Protocols.io 10.17504/protocols.io.bi8zkhx6). To allow for multiplexing, gDNA was barcoded using IDT-ILMN Nextera DNA UD Indices, a set of 10 bp index adapters from Illumina. Indexed samples were diluted to 4 nM, pooled, and analyzed on an Agilent TapeStation to ensure the mean DNA fragment size was ~300 bp. Pooling and library quality was further assessed by sequencing the pool using a V3 MiSeq flow cell. 160 samples were pooled and sequenced for 76 cycles, paired end reads. For the purpose of QC, ~50 million reads were obtained and Q30 was determined to be >92%. If needed the pool was normalized (balanced) to ensure equal representation of each sample. The library was then sequenced on an Illumina NovaSeq 6000 using an S4 300 cycle flow cell.

### Viral RNA library preparation and sequencing

After extraction, RNA acquired from 100 ul nasal swab media was incubated with recombinant RNAse-free DNase (Qiagen, Inc.) per manufacturer’s instructions for 15 minutes, followed by SPRI bead (GE Healthcare) purification to remove residual DNA remaining in each sample. A fixed volume (5uL) of the resulting RNA from each sample, together with a fixed mass (25 pg) of the External RNA Controls Consortium RNA spike-in mix (ERCC RNA spike-in mix, Thermo Fisher), served as input for SARS-CoV-2 metatranscriptomic next generation sequencing (mNGS) library preparation (10.17504/protocols.io.beshjeb6; a modification of Deng et al.^32^).

For samples collected after May 2020, SARS-CoV-2 ARTIC V3 amplicon libraries were made from extracted total nucleic acid for whole genome sequencing using previously reported protocols ^[1]^. Briefly, 3 ul of total nucleic acid was used as input for a randomly primed cDNA synthesis reaction. This cDNA served as input for 30 cycles of amplification with ARTIC V3 primers (https://github.com/artic-network/artic-ncov2019), and was then diluted 1:100 before tagmentation. Adaptor tagmentation was performed using homebrew Tn5, and 8 cycles of index PCR was performed using unique dual barcode Nextera indices (Detailed protocol: https://protocols.io/view/artic-neb-tagmentation-protocol-high-throughput-wh-bt66nrhe). Final libraries were pooled at equal volumes and cleaned at 0.7x (SPRI: Sample) using SPRIselect beads. Library was sequenced on Illumina Novaseq SP platform in a paired-end 2 ×150 cycle run. An incubation step with 1:10 dilution of FastSelect (Qiagen) reagent was included between the RNA fragmentation and first strand synthesis steps of the library prep to deplete highly abundant host rRNA sequences present in each sample. Equimolar pools (*n* = 160–384 samples) of the resulting individual dual-barcoded library preps were subjected to paired-end 2 x 150 bp sequence analysis on an Illumina NovaSeq 6000 (S2 or equivalent flow cell) to yield approximately 50 million reads per sample.

### Viral and metagenomic alignment and metagenomics analysis

For SARS-CoV-2 genomes, FASTQ sequences were aligned to the SARS-CoV-2 reference genome NC_045512.2 using minimap2^33^. Non-SARS-CoV-2 reads were filtered out with Kraken2^34^, using an index of human and viral genomes in RefSeq (index downloaded from https://genexa.ch/sars2-bioinformatics-resources/). Spiked primers for viral enrichment were trimmed from the ends of short reads using ivar^35^. Finally, a pileup of the aligned reads was generated with samtools^36^, and consensus genomes were called with ivar. The full pipeline used is publicly available on Github (https://github.com/czbiohub/sc2-illumina-pipeline). All viral consensus sequences were uploaded to the GISAID database (https://www.gisaid.org/).

Host and metagenomic RNA alignment was performed using STAR run against a combined index of the human reference genome GRCh38, SARS-CoV2 (SARSCoV2_NC_045512.2), and ERCC spike-ins. STAR parameters were chosen to avoid bias towards GTAG eukaryotic splice signatures for both the viral RNA and host RNA analyses. Metagenomic classification of reads unmapped to both SARS-CoV2 and human was performed using KrakenUniq^37^. KrakenUniq parameters (>=100 kmers and duplication < = less kmers) were chosen to avoid false positives. From the filtered KrakenUniq output, an abundance table was created by finding the kmer percentages (kmers divided by the total kmer count) for relevant taxa detected for each individual. This table included only well-represented taxa, which was defined as those appearing in at least 10% of patients. A uniform manifold approximation (UMAP) plot was then created from this table using fifteen nearest neighbors. In order to identify associations between specific microbial species and degree of severity of COVID symptoms for each patient, we used a linear regression of severity against each species’ abundance separately and used BMI, sex, and age as covariates of the analysis. The significance of the association was thresholded at a Bonferroni adjusted p-value of 7e-04.

### Host genome sequence alignment

Low-coverage FASTQ sequences underwent quality control assessment via FastQC v0.11.8 before alt-aware alignment to GRCh38.p12 using BWA-MEM v0.7.17-r1188. Duplicate sequences were marked with MarkDuplicates of the Picard Tools suite v2.21.2. After duplicate marking, base quality score recalibration was performed with Picard Tools’ BaseRecalibrator and high-confidence variant call sets from dbSNP and the 1000 Genomes Project. Quality control metrics, including coverage, were generated with Qualimap BAMQC v2.2.1, Samtools v1.10, and Mosdepth v0.2.9. Finally, quality control reports for each sample were aggregated using MultiQC v1.9. Reproducible code and steps are available at Protocols.io (https://www.protocols.io/private/8CFBD1AD8FE611EA815E0A58A9FEAC2A). All high confidence calls were contributed to the COVID-19 Host Genetics Initiative^3^.

### Variant calling, imputation, PCA, kinship

BAM files were used for an initial calling with bcftools v1.9 mpileup^38^. To account for the low-coverage sequencing we used the GLIMPSE algorithm v1.0 for imputation and phasing^15^. Briefly, this algorithm uses a reference set of haplotypes (1000 Genomes Project samples in our case) to compute genotype likelihoods using a Gibbs sampling procedure. The imputed data were filtered for low imputation scores (INFO > 0.8), and were then merged with a reference set that contained samples from: (1) the 1000 Genomes Project^39^, (2) the Human Genome Diversity Project (HGDP)^40^, and (3) the Simons Genome Diversity Project (SGDP)^41^. Pre-filtering, there were 1194 samples in our patient data set, and 3558 in the reference set. 1062 remained in our patient cohort after sample QC, and from the reference set 1359 samples were used for ancestry analyses. While merging these data, we set minor allele count (MAC) thresholds for our data at 2 (MAF 0.0008) and for the reference set at 5 (MAF 0.0007) (e.g., MAC > 4 using bcftools), and a stringent call rate threshold (*--geno 0.01* in PLINK2)^42,43^. The resulting VCF was loaded into PLINK2 v2.00a3LM using the following flags: *dosage* = *DS, --import-dosage-certainty 0.8*. These merged data had 4,111,339 autosomal variants that survived the filters above. PLINK2 was then used for LD pruning (*--indep-pairwise 500 10 0.1*) and PCA (*--maf 0.01 --pca*). We also extracted the kinship matrix of our samples using the King algorithm (*--make-king* in PLINK2)^44^. Missingness data by chromosome is available in Supplementary Data 3. For admixture mapping, all individuals with third degree (cousins) or lower relatives in the dataset were removed.

### Genetic Ancestry Inference and majority-vote assignation

Genetic ancestry was determined by running supervised local ancestry inference (RFMix v2.03)^45^ on the above phased and imputed patient genomes using a training reference panel of single ancestry samples selected from the 1000 Genomes Project, HGDP, and SGDP via unsupervised genetic clustering (ADMIXTURE)^46^ at *K* = 7 (*N* = 1359). Only individuals with greater than 0.95 assignment to one of the seven unsupervised clusters in that ADMIXTURE analysis were used as references for RFMix. The cluster labels--African (AFR), East Asian (EAS), South Asian (SAS), Oceanian (Australo-Papuan) (OCE), European (EUR), West Asian (WAS), and Indigenous American (NAT)--were chosen to reflect the biogeographic origin of the reference samples found in each unsupervised cluster. The number of individuals thus included in the RFMix reference training are as follows: AFR- 382, EAS- 494, EUR- 155, NAT- 75, OCE- 16, SAS- 171, WAS- 66. Local genetic ancestry assignments along the genome were then summed to create overall genetic ancestry proportions for each sample. These were used for barplots, covariates for regression analyses, and for making individual genetic ancestry assignments. Individual genetic ancestry labels (e.g., for determination of enrichment in cases vs. controls (Fig. 1F), association with viral clades (Fig. 1H) and controlling HLA associations with severe disease by genetic ancestry) were assigned based on these overall proportions via the following decision sequence: some Oceanian (Australo-Papuan) ancestry (Pacific Islanders)^47^ >5%, some Indigenous American ancestry >10%, West Asian >50%, South Asian >50%, East Asian >50%, European >50%, African >50%. For individuals meeting none of these criteria an ancestry label consisting of the two predominant ancestries was given (e.g., East Asian and European in Fig. 1F). 

### Admixture mapping association analyses

Admixture mapping association analyses were used to regress the residual of severity of COVID symptoms for each patient--after correcting for associations with overall genetic ancestry proportion, BMI, sex, and age--against the local ancestry of each particular window of the genome for that patient^48^. With the genome subdivided into 19,474 windows for local genomic ancestry assignment, and assuming complete independence between each, a naive Bonferroni corrected p-value of 2.57*10^−6 is obtained for genome-wide significance at *p* = 0.05; however, the genomic ancestry of neighboring, linked genomic windows is not independent and depends upon the characteristic length of each ancestry segment distribution, itself a function of the time since admixture in each population. A less stringent multiple-test correction factor that incorporates this distribution was determined by considering the spectral density evaluated at frequency zero of an autoregressive model of local ancestries^19^, yielding an effective number of tests for each ancestry. This overall effective number of tests was taken over only samples that had at least 5% of that ancestry represented across their autosomes. Using this framework, together with the spectrum0.ar function implemented in the R package coda v 0.19, p-value thresholds for genome-wide significance at *p* = 0.05 for each ancestry were determined: African 2.54*10^−3, East Asian 3.89*10^−3, Indigenous American 1.15*10^−3, Oceanian 6.93*10^−3, South Asian 2.21*10^−3, and European/West Asian 1.83*10^−3. Variants assessed as significant by this correction are described in Supplementary Data 1. An additional analysis was performed in which case and control labels were randomly permuted amongst the samples to generate 1000 separate datasets, association analyses were then performed on each of these replicate datasets. To obtain a study-specific null distribution, the lowest *p* value for each of these permuted replicates was recorded, and a study-specific p-value threshold (.05 quantile of this aggregate distribution of minimal *p* values) was obtained for each ancestry: African 5.39*10^−4, East Asian 6.1*10^−3, Indigenous American 1.84*10^−3, Oceanian 2.05*10^−6, South Asian 5.3*10^−4, and European/West Asian 1.73*10*−2. Two associations are significant under both of these thresholds: an association on chromosome 14 (43962800-44734273, p-value 1.6e−08) with Oceanian ancestry and an association on chromosome 21 (36748417-38748006, *p*-value 5.1e−04) with African ancestry.

### Host HLA sequencing and typing

Host genomic DNA samples ranging from 22-75 ng were batched in sets of 46 plus one positive and one negative control. AllType™ FASTplex™ NGS Assay kits (One Lambda, A Thermo Fisher Scientific Brand, Canoga Park, CA) were used to prepare DNA sequencing libraries for 11 classical HLA genes (*HLA-A*, *HLA-C*, *HLA-B*, *HLA-DRB3*, *HLA-DRB4*, *HLA-DRB5*, *HLA-DRB1*, *HLA-DQA1*, *HLA-DQB1*, *HLA-DPA1*, and *HLA-DPB1*). As the success of the DNA sequencing is dependent on the initial target amplification and the subsequent library preparation, the following changes were made to the manufacturer’s protocol: (1) increased input DNA volume to 8.6 µl while maintaining the manufacturer’s recommended multiplex PCR protocol per sample; (2) eluted DNA in 12 µl of suspension buffer after the initial amplicon purification, and proceeded to the library preparation without normalization process; (3) increased the number of thermal cycles to 17 in the final DNA library amplification; (4) eluted DNA fragments in 22 µl for DNA sequencing. 500 µl of 1.3 pM DNA sequencing library was loaded into a MiniSeq Mid Output Kit (300-cycles) (FC-420-1004), and sequenced using MiniSeq DNA sequencer (Illumina Inc., San Diego, CA).

A total of 429 subjects (301 cases, 128 SARS-CoV-2 negative controls) yielded interpretable sequence reads to generate HLA genotypes. We supplemented these samples with sequencing of buffy coat or whole blood collected from high severity COVID-19 patients collected from hospitalized patients (*n* = 193). Fastq files were automatically imported into the TypeStream Visual NGS Analysis Software Version 2.0 upon the completion of DNA sequencing, and bioinformatically processed for DNA sequence assembly and HLA genotype assignments with IPD IMGT/HLA Database release version 3.39.0^49^. We modified the software setting so that a maximum of 1.5 million sequences or 750,000 paired-end sequences are used for the sequence assembly and HLA allele assignments. We visually inspected the HLA genotype calls by the software, and made corrections as needed. The approved HLA genotype results were exported in Histoimmunogenetics Markup Language (HML) format^50^, and generated comma separated value (CSV) reports for HLA genotypes, HLA serotypes including Bw4 and Bw6, KIR ligands (C1 and C2) and imputed HLA haplotypes^51,52^.

Subjects were grouped in three categories (Negative: SS_MAX = 0; Mild: SS_MAX = 1 –3; Severe: SS_MAX = 4 −8), and organized in six broad ancestry groups [European (EUR), Hispanic (HIS), Asian (ASI), African American (AFA), Native American (NAM) and Native Hawaiian/Pacific Islander (HPI)] based on self-reported ethnicity in clinical records. When self-reported ethnicity was not available, genetic ancestry calculated from the low pass WGS in this study was used as described above. We converted the genetic ancestry information to self-reported medical record ethnicity format as follows: European and West Asian =>  EUR; some Indigenous American =>  HIS; East Asian and South Asian => ASI; African =>  AFA; fully Indigenous American =>  NAM; some Oceanian =>  HPI. We compared the distribution of both HLA serotypes and alleles from COVID-19+ individuals with low disease severity (maximum severity score 1−3, *n* = 336) to those with high disease severity (maximum severity score 4-8, *n* = 94). HLA serotype and allele frequencies were calculated in both Mild and Severe groups, and Odds Ratio (OR: Mild vs. Severe) and *p*-values were calculated for each serotype and allele using Bridging ImmunoGenomic Data-Analysis Workflow Gaps (BIGDAWG)^53^. Cochran- Mantel-Haenszel (CMH) tests^54^ were subsequently performed for all observed HLA-A, -B, -C, -DRB1, -DQB1 and -DPB1 serotypes and alleles across three major ethnic groups (EUR, HIS and ASI) using the “mantelhaen.test” function in stats R package. Subjects with AFA, NAM and HPI ethnic groups were excluded from CMH tests, because we had only 6, 1 and 5 subjects, respectively, that yielded HLA genotypes.

### Phylodynamic analysis

For Bayesian inference of the viral phylogeny, we assumed the Extended Bayesian Skyline Plot^55^ prior on the effective population size and coalescent prior on the phylogeny, a fixed molecular clock with a uniform prior distribution centered at 8 × 10^−4^ substitutions per site per year as done in^56^. We assumed the HKY mutation model^57^ with default hyperparameter priors in the BEAST2 software^58^. We ran a Markov chain Monte Carlo chain to approximate the posterior distribution of the model parameters for 20 million iterations and thinned every 5000 iterations. The first 10% of samples were discarded as burn-in. We used Tracer^59^ to assess the convergence and confirm that the effective sample size (ESS) was >120 for all parameters (except in 15% of effective population size parameters, estimations not shown). Finally, we used TreeAnnotator^60^ to summarize the phylogeny posterior distribution and generated the maximum clade credibility tree of Fig. 1H. To test the association between clade composition and binary traits, we used the R package treeSeg^61^.

### Reporting summary

Further information on research design is available in the Nature Research Reporting Summary linked to this article.

## Supplementary information

Supplementary Information

Description of Additional Supplementary Files

Supplementary Data 1

Supplementary Data 2

Supplementary Data 3

Reporting Summary

## Data Availability

As is described above, the data generated in this study have been deposited at https://covid-omics.org/results. Raw sequencing and clinical data are not available based on requirements for anonymity and de-identification as outlined in internal review board approval. Consensus viral sequences have been uploaded to GISAID. Imputed Genomic data were filtered for low imputation scores (INFO > 0.8), and were then merged with a reference set that contained samples from: (1) the 1000 Genomes Project (https://www.internationalgenome.org/data), (2) the Human Genome Diversity Project (HGDP, https://www.internationalgenome.org/data-portal/data-collection/hgdp), and (3) the Simons Genome Diversity Project (SGDP, https://www.simonsfoundation.org/simons-genome-diversity-project/). HLA calls were made against IPD IMGT/HLA Database release version 3.39.0.

## Code availability

Custom code for viral sequence alignment is available on GitHub: https://github.com/czbiohub/sc2-illumina-pipeline.
