# Supplementary material for: Deconvoluting complex correlates of COVID-19 severity with a multi-omic pandemic tracking strategy
Source: Nat Commun. 2022 Aug 30;13:5107. doi: 10.1038/s41467-022-32397-8 (PMC9426371; doi:10.1038/s41467-022-32397-8)
Supplement: Supplementary file 1 — Supplementary Information [file 41467_2022_32397_MOESM1_ESM.pdf]

Supplementary Material

Deconvoluting complex correlates of COVID19 severity with a multi-omic pandemic tracking strategy

Victoria N. Parikh, MD[1]*; Alexander G. Ioannidis, PhD[2,3]*; David Jimenez-Morales, PhD[1]; John E. Gorzynski, DVM[1,4]; Hannah N. De Jong, PhD[1,4]; Xiran Liu, MS[3]; Jonasel Roque, BS[1]; Victoria P. Cepeda-Espinoza, PhD[2]; Kazutoyo Osoegawa, PhD[5]; Chris Hughes, BS[1,4]; Shirley C. Sutton, MS[1,4]; Nathan Youlton, BS[1,4]; Ruchi Joshi, MS[1]; David Amar, PhD[1]; Yosuke Tanigawa, PhD[2]; Douglas Russo, MS[6]; Justin Wong, MS[6]; Jessie T. Lauzon, MS[7]; Jacob Edelson, MS[2]; Daniel Mas Montserrat, PhD[2]; Yongchan Kwon, PhD[2]; Simone Rubinacci, PhD[8]; Olivier Delaneau, PhD[8]; Lorenzo Cappello, PhD[6]; Jaehee Kim, PhD[9]; Massa J. Shoura, PhD[4,10]; Archana N. Raja, PhD[1]; Nathaniel Watson[10]; Nathan Hammond[10]; Elizabeth Spiteri PhD[10]; Kalyan C. Mallempati[5]; Gonzalo Montero-Martín, PhD[5]; Jeffrey Christle, PhD[1]; Jennifer Kim, BS[1]; Anna Kirillova, BS[11]; Kinya Seo, PhD[1]; Yong Huang, MD[1]; Chunli Zhao, PhD[1]; Sonia Moreno-Grau PhD[2]; Steven G. Hershman, MS, MSE, PhD[1]; Karen P. Dalton[1]; Jimmy Zhen[1]; Jack Kamm, PhD[12], Karan D. Bhatt, MS[12]; Alina Isakova, PhD[13]; Maurizio Morri, PhD[12]; Thanmayi Ranganath, MS[1]; Catherine A. Blish MD PhD[1]; Angela J. Rogers, MD[1]; Kari Nadeau, MD PhD[1,14]; Samuel Yang, MD[15]; Andra Blomkalns, MD[15]; Ruth O'Hara, MD PhD[16]; Norma F. Neff, PhD[12]; Christopher DeBoever, PhD[2], Sándor Szalma[17], Matthew T. Wheeler MD PhD[1]; Christopher Gates[18]; Kyle Farh, MD PhD[18]; Gary P. Schroth, PhD[18], Phil Febbo, MD[18], Francis deSouza[18], Omar E. Cornejo, PhD[19], Marcelo Fernandez-Vina, PhD[5,10]; Amy Kistler, PhD[12]; Julia Palacios, PhD[2,6]; Benjamin A. Pinsky, MD PhD[1,10]; Carlos D. Bustamante, PhD[2]*; Manuel A Rivas, PhD[2]*; Euan A. Ashley, MD PhD[1,4]*

Supplementary Note 1

Additional detail regarding HLA-Severity associations

The *HLA-C*15:02* allele is common in some Native American tribes from North, Central and South America. The alleles of *HLA-C* can be classified into the C2 and C1 groups according their interaction with the inhibitory receptors KIR2DL1 and KIR2DL2/3 present in NK cells, respectively; the allele *HLA-C*15:02* belongs to the C2 group. The current study found no significant associations of other *HLA-C* alleles of the C2 group or of other HLA ligands of KIR receptors suggesting that the broad KIR/HLA ligand interactions are not major determinants of disease course. We could not confirm the risk reduction in the prototype allele *HLA-B*15:01* for the serotype B62, because we observed other B62 alleles (*HLA-B*15:04*, *-B*15:25*, *-B*15:27*, *-B*15:35*, *-B*15:48* and *-B*15:146*). They are mostly HLA alleles for Hispanic and Asian. The *HLA-B*15:01* allele was recently associated with asymptomatic SARS-CoV-2 infection in European ancestry subjects. In our study subjects only 32% percent of the subjects had European ancestry; we did not find statistical significance for the individual allele *HLA-B*15:01* that is by far more common than other alleles of the serotype B62 in Caucasians. The alleles *HLA-B*15:04*, *-B*15:25*, *-B*15:27*, *-B*15:35*, *-B*15:48* and *-B*15:146* are found in subjects with Hispanic and Asian. The B*15 alleles corresponding to the B62 serotype detected in the present study show high structural homology (differing by 1-5 amino acid replacements) and share identity in the B-pocket substructure that determines what type of peptide residue at the second position is anchored. A recent study in outcomes of hematopoietic stem cell transplantation found that the HLA-B62 supertype was associated with decreased transplant-related mortality in the entire patient cohort.[62] It can be speculated that HLA-B62 may play a role in immune response; the associations identified in the present study lend support to the hypothesis that peptide presentation by alleles of the B62 serotype in addition to the *HLA-C*15:02* allele may contribute to beneficial outcomes in SARS-CoV-2 Disease.

Supplementary Tables

Supplementary Table 1. WHO-based COVID-19 Clinical Severity Scale

**Score**	**Patient Status**	**Description**	**EHR annotations**
0	Uninfected	No clinical or virological evidence of infection	SARS-CoV-2 negative
1	Ambulatory	Asymptomatic	Non COVID-related symptoms
2		Symptomatic	COVID related symptoms (cough, fever, shortness of breath, chest pain, tachycardia, acute pharyngitis, fever, others)
3	Hospitalized, Mild disease	Hospitalized, no oxygen	COVID related symptoms + hospitalization time (without oxygen support)
4		Hospitalized, oxygen mask	Nasal cannula; face mask; high-oxygen mask; aerosol mask; tracheal collar; others
5	Critical Care, Severe disease	non-invasive ventilation / high flow oxygen	High-flow nasal cannula; non-invasive positive pressure ventilation
6		intubation and mechanical ventilation	Invasive Ventilation; endotracheal tube
7		intubation + organ support	Intubation + dobutamine, dopamine, epinephrine, norepinephrine, phenylephrine, vasopressin
8	Dead	Deceased	Deceased

Supplementary Figures

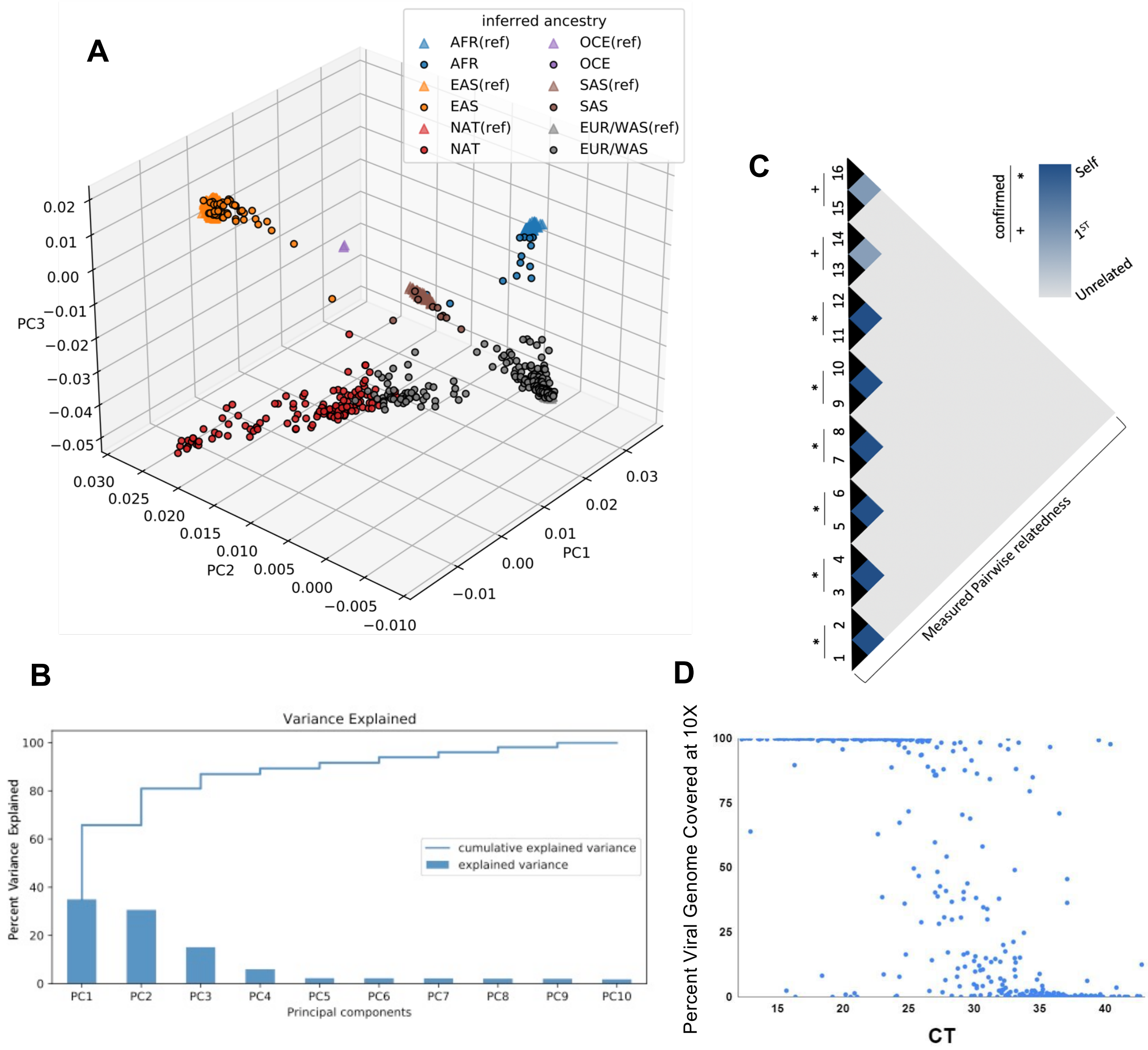

Supplementary Figure 1. (A) Variance explained by the top 10 PCs. The first four PCs explain ~90% of variance. **(B)** Genotyping by low pass WGS recapitulates known kinship relationships **. (C)** Genomic data of patients (bold points) projected onto top three PCs created by samples from 1000 Genomes, HGDP and SGDP (light points). **((D)** Viral genome recovery using ARTIC primer capture was improved at higher CT values compared with shotgun RNAseq (Figure 1D).

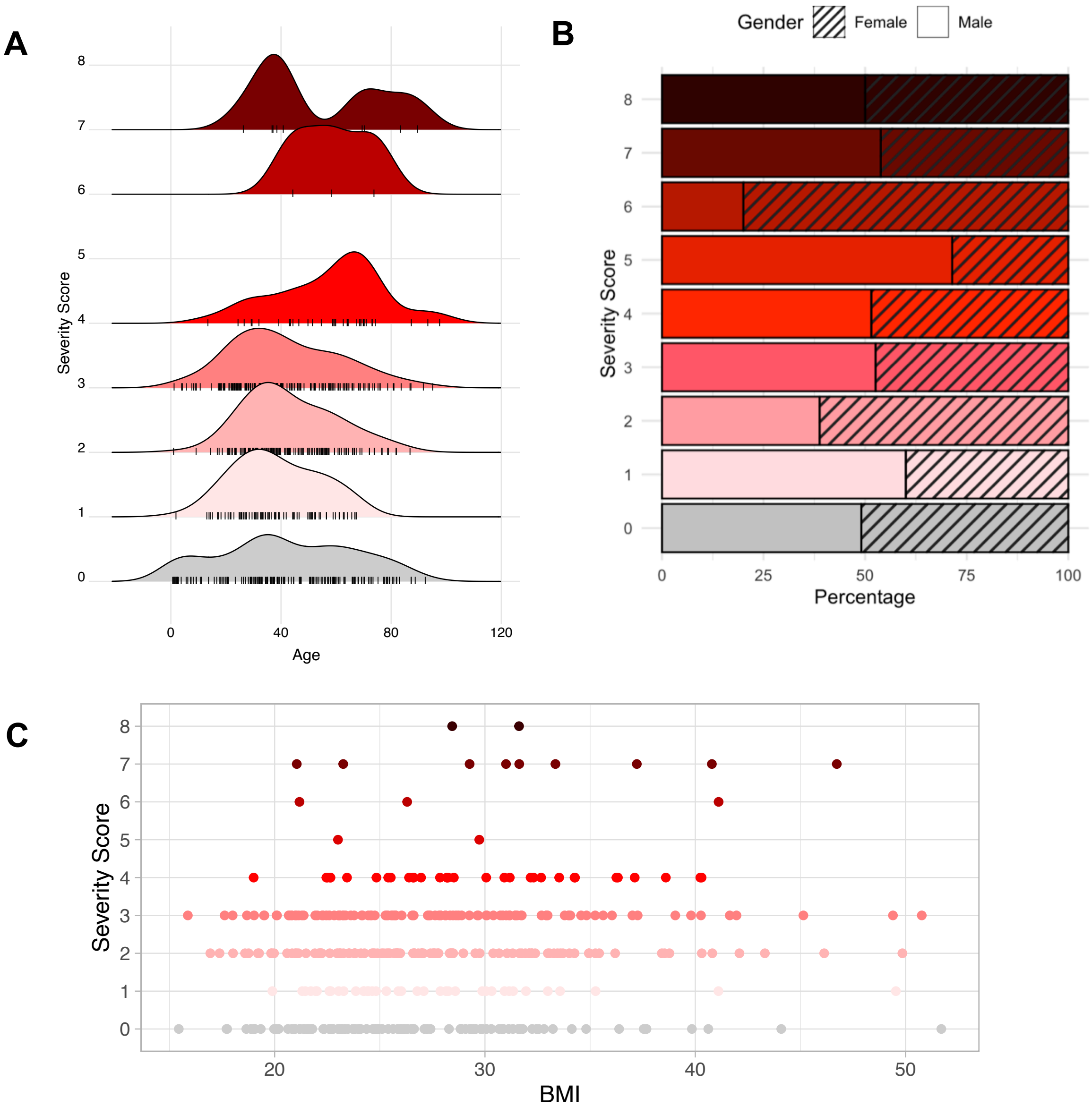

Supplementary Figure 2. Characteristics of COVID19 positive individuals versus negative controls stratified by severity (I). (A) Distributions of patient age by severity score. **(B)** Distribution of sex by severity score. **(C)** Distribution of BMI by severity score.

A

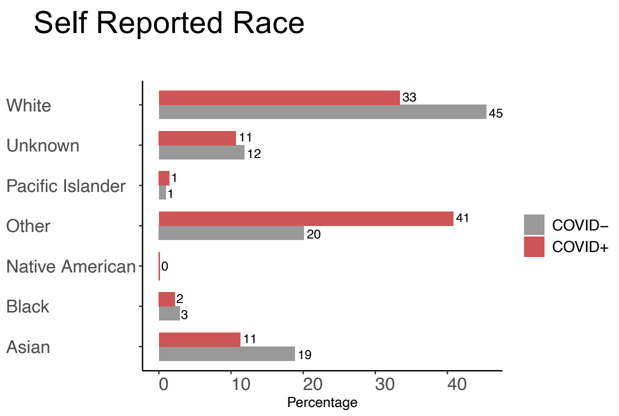

B

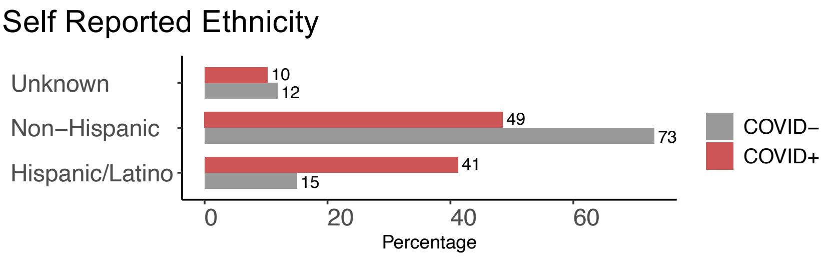

C

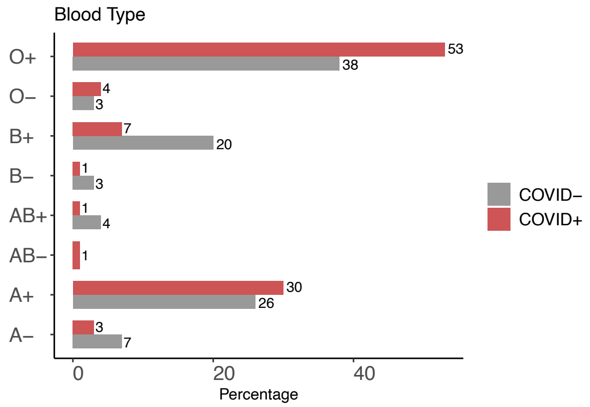

D

HLA	OR [95% CI]	p-value
B7	2.1 [1.1, 3.9]	2.32E-02
*B*07:02*	2.7 [1.4, 5.1]	2.89E-03
Cw15	0.3 [0.09, 0.96]	4.66E-02
*C*15:02*	0.12 [0.02, 0.82]	1.41E-02
A31	2.5 [1.2, 5.1]	1.99E-02
*A*31:01*	2.7 [1.3, 5.5]	1.17E-02
B62	0.23 [0.05,0.96]	4.76E-02

Supplementary Figure 3. Characteristics of COVID19 positive individuals versus negative controls (II). (A) Self reported race. **(B)** Self Reported Ethnicity, **(C)** Blood type. **(D)** HLA allele and serotype (italics) association with COVID19 severity after ancestry adjustment by Cochran Mantel Haenszel test. P-values (two-sided) presented are not corrected for multiple testing.

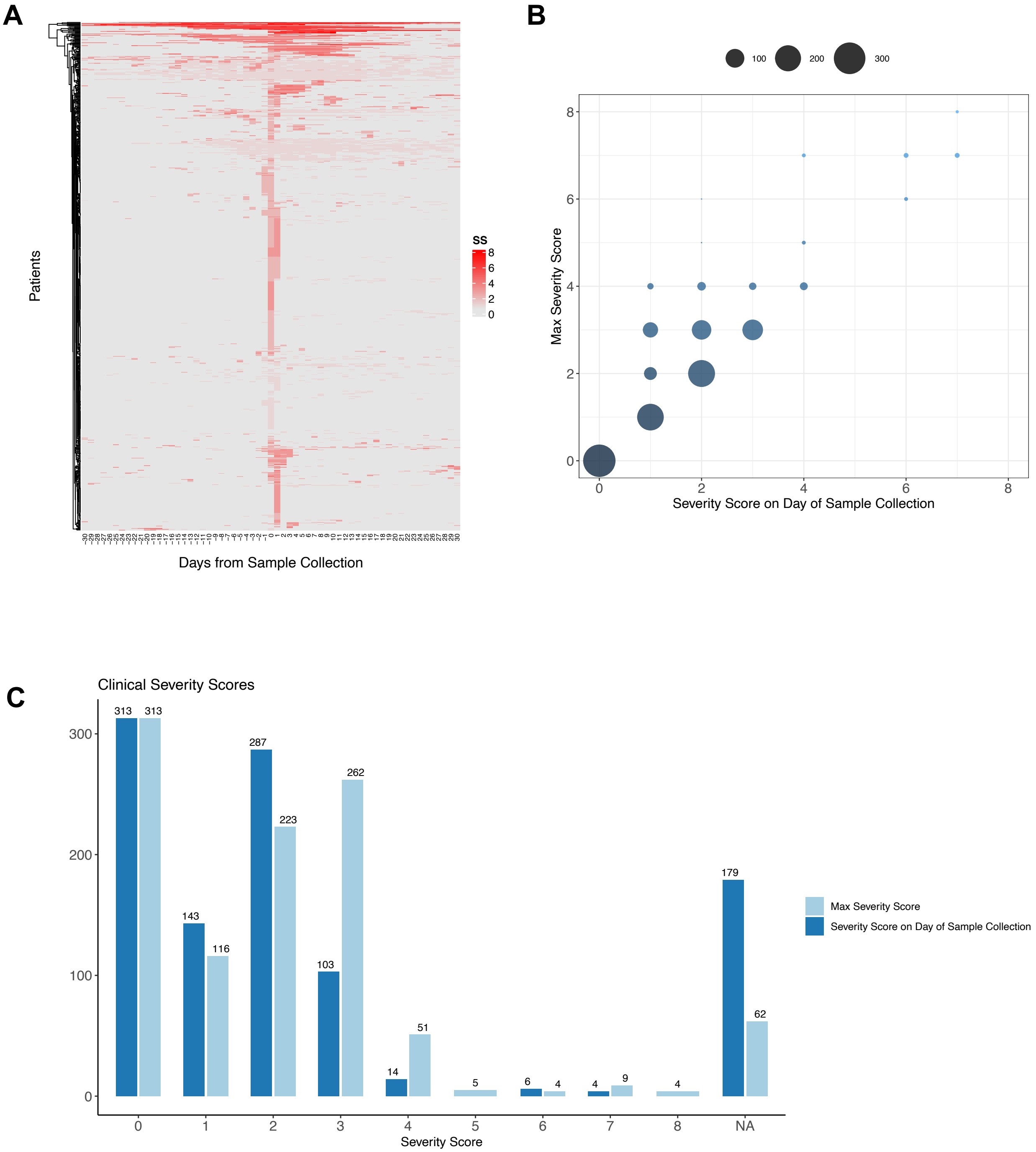

Supplementary Figure 4. Severity score. (A) Variance in severity score for 30 days before and after NP swab collection for all samples. (B) Correlation between severity on day of sample collection and highest severity (Max Severity Score) in the 30 days before and after swab collection. (C) Redistribution of severity scores in the 30 days before and after collection vs day of collection.

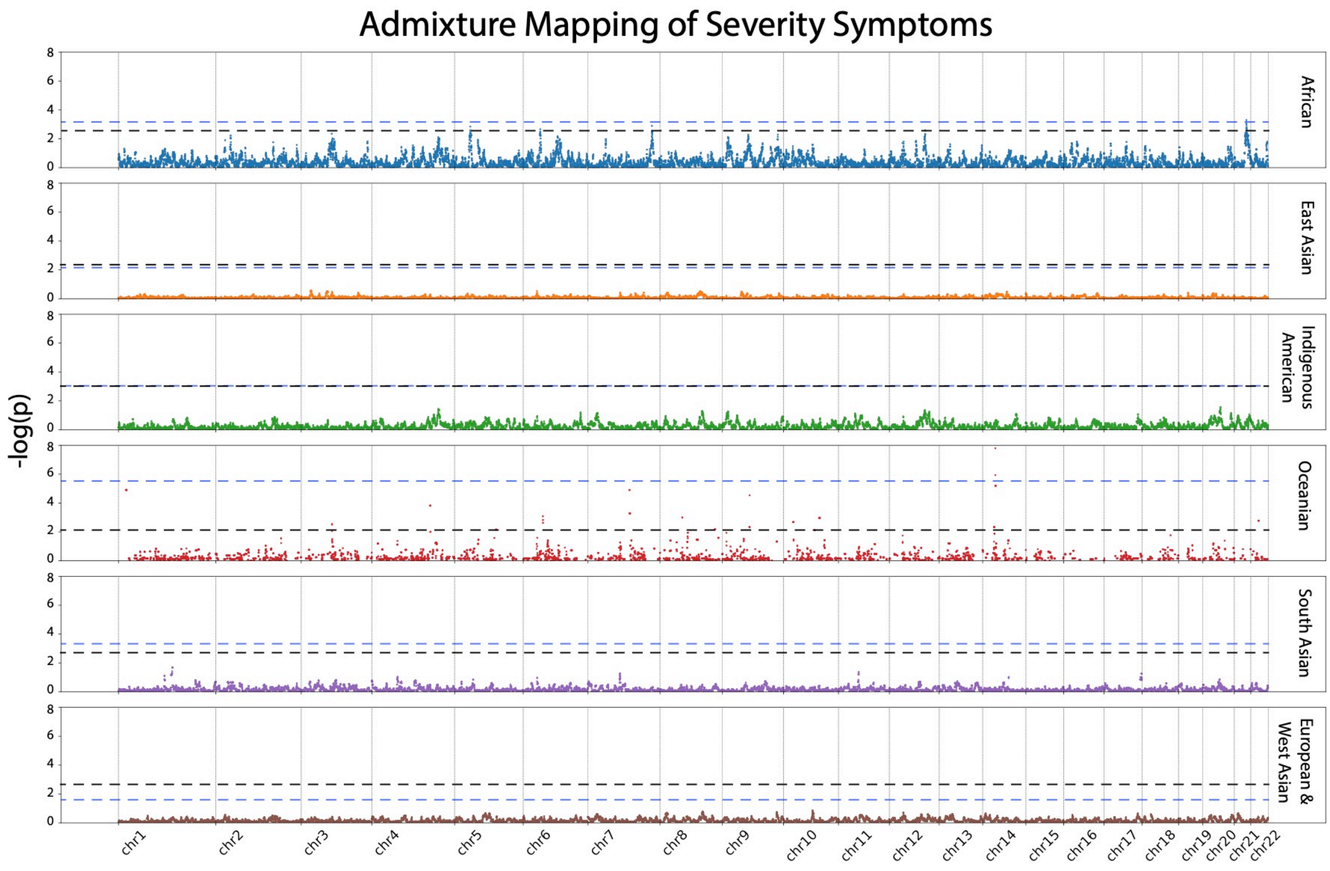

Supplementary Figure 5. Admixture mapping to COVID19 severity displaying significance thresholds. Ancestry-specific risk loci found in African and Oceanian ancestries, respectively. Each dot represents a window of the genome. Black lines represent ancestry-specific thresholds determined by the method of Shriner et al. Blue lines represent thresholds determined by running one thousand association tests on random permutations of case-control labels as described in Online Methods.
