# Supplementary material for: Deconvoluting complex correlates of COVID-19 severity with a multi-omic pandemic tracking strategy
Source: Nat Commun. 2022 Aug 30;13:5107. doi: 10.1038/s41467-022-32397-8 (PMC9426371; doi:10.1038/s41467-022-32397-8)
Supplement: Supplementary file 2 — Description of Additional Supplementary Files [file 41467_2022_32397_MOESM2_ESM.pdf]

Description of Additional Supplementary Files

File Name: Supplementary Data 1
Description: Summary of GWAS catalog hits in genomic regions associated with COVID19 severity using admixture mapping.

File Name: Supplementary Data 2
Description: Patient characteristics by analysis.

File Name: Supplementary Data 3
Description: Missingness report.
