# Supplementary material for: Deconvoluting complex correlates of COVID-19 severity with a multi-omic pandemic tracking strategy
Source: Nat Commun. 2022 Aug 30;13:5107. doi: 10.1038/s41467-022-32397-8 (PMC9426371; doi:10.1038/s41467-022-32397-8)
Supplement: Supplementary file 6 — Reporting Summary [file 41467_2022_32397_MOESM6_ESM.pdf]

nature portfolio

Corresponding author(s): Victoria Parikh and Euan Ashley

Last updated by author(s): Jul 13, 2022

Reporting Summary

Nature Portfolio wishes to improve the reproducibility of the work that we publish. This form provides structure for consistency and transparency in reporting. For further information on Nature Portfolio policies, see our Editorial Policies and the Editorial Policy Checklist.

Statistics

For all statistical analyses, confirm that the following items are present in the figure legend, table legend, main text, or Methods section.

n/a	Confirmed	
☐	☒	The exact sample size (*n*) for each experimental group/condition, given as a discrete number and unit of measurement
☒	☐	A statement on whether measurements were taken from distinct samples or whether the same sample was measured repeatedly
☐	☒	The statistical test(s) used AND whether they are one- or two-sided<br>*Only common tests should be described solely by name; describe more complex techniques in the Methods section.*
☐	☒	A description of all covariates tested
☐	☒	A description of any assumptions or corrections, such as tests of normality and adjustment for multiple comparisons
☐	☒	A full description of the statistical parameters including central tendency (e.g. means) or other basic estimates (e.g. regression coefficient) AND variation (e.g. standard deviation) or associated estimates of uncertainty (e.g. confidence intervals)
☐	☒	For null hypothesis testing, the test statistic (e.g. *F*, *t*, *r*) with confidence intervals, effect sizes, degrees of freedom and *P* value noted<br>*Give P values as exact values whenever suitable.*
☐	☒	For Bayesian analysis, information on the choice of priors and Markov chain Monte Carlo settings
☒	☐	For hierarchical and complex designs, identification of the appropriate level for tests and full reporting of outcomes
☒	☐	Estimates of effect sizes (e.g. Cohen's *d*, Pearson's *r*), indicating how they were calculated

Our web collection on statistics for biologists contains articles on many of the points above.

Software and code

Policy information about availability of computer code

Data collection	No software or custom code was used for data collection.
Data analysis	The following publicly available software was used as referenced in the methods section: R, GLIMPSE algorithm v1.0, PLINK2, RFMix v2.03, TypeStream Visual NGS Analysis Software Version 2.0, Bridging ImmunoGenomic Data-Analysis Workflow Gaps (BIGDAWG), BEAST2, Tracer, and TreeAnnotator.

For manuscripts utilizing custom algorithms or software that are central to the research but not yet described in published literature, software must be made available to editors and reviewers. We strongly encourage code deposition in a community repository (e.g. GitHub). See the Nature Portfolio guidelines for submitting code & software for further information.

Data

Policy information about availability of data

All manuscripts must include a data availability statement. This statement should provide the following information, where applicable:

- Accession codes, unique identifiers, or web links for publicly available datasets
- A description of any restrictions on data availability
- For clinical datasets or third party data, please ensure that the statement adheres to our policy

Imputed genomic data were were filtered for low imputation scores (INFO>0.8), and were then merged with a reference set that contained samples from: (1) the 1000 genomes project, (2) the Human Genome Diversity Project (HGDP),38 and (3) the Simons Genome Diversity Project (SGDP) and (4) IPD IMGT/HLA Database release version 3.39.0. As is described above, the data generated in this study have been deposited at https://covid-omics.org/results. Raw sequencing and clinical data are not available to due to privacy laws. Consensus viral sequences have been uploaded to GISAID.

Field-specific reporting

Please select the one below that is the best fit for your research. If you are not sure, read the appropriate sections before making your selection.

- [x] Life sciences
- [ ] Behavioural & social sciences
- [ ] Ecological, evolutionary & environmental sciences

For a reference copy of the document with all sections, see nature.com/documents/nr-reporting-summary-flat.pdf

Life sciences study design

All studies must disclose on these points even when the disclosure is negative.

Sample size	Residual viral transport media (VTM) samples from SARS-CoV-2 clinical diagnostic tests were prospectively collected from March 2020 to July 2020 from Stanford Health Care in Northern California, USA. Swabs were selected approximately consecutively from SARS-CoV-2 positive and negative individuals and linked to structured clinical information from the electronic health record. Sample size was not pre-specified in this pandemic-tracking design, and given the wide range of studies performed, variable statistical power was available based on the number of included subjects as described in Supplementary Data 2.
Data exclusions	Detailed data inclusion criteria are described for each analysis in online methods. Supplemental File 2 shows that distributions of severity age sex and BMI for individuals included in each analysis.
Replication	Internal replication of our findings was not possible given we were only able to collect one prospective dataset of SARS-CoV-2 infected individuals. For validation, we compared our findings to prior GWAS studies (eg the COVID19 host genetics consortium) and results in the GWAS catalog, and for variant calls and RFMix by comparing to a reference data set as described.
Randomization	Randomization was not applicable to this prospective enrollment cohort study design. There was no intervention to randomize to.
Blinding	Data collection was performed by different team members than those who did the analysis. Blinding was not performed for analysts given large sample size and lack of subjectivity inherent to software used for analysis.

Reporting for specific materials, systems and methods

We require information from authors about some types of materials, experimental systems and methods used in many studies. Here, indicate whether each material, system or method listed is relevant to your study. If you are not sure if a list item applies to your research, read the appropriate section before selecting a response.

Materials & experimental systems

n/a	Involved in the study	
☒	☐	Antibodies
☒	☐	Eukaryotic cell lines
☒	☐	Palaeontology and archaeology
☒	☐	Animals and other organisms
☐	☒	Human research participants
☒	☐	Clinical data
☒	☐	Dual use research of concern

Methods

n/a	Involved in the study	
☒	☐	ChIP-seq
☒	☐	Flow cytometry
☒	☐	MRI-based neuroimaging

Human research participants

Policy information about studies involving human research participants

Population characteristics	Please see supplemental file 2 for detailed population characteristics.
Recruitment	Approximate consecutive selection of discarded VTM by the clinical laboratory as detailed.
Ethics oversight	This work was performed under protocol IRB-55580, which was approved by the Stanford University School of Medicine IRB and its most recent approval was 5/6/2021.

Note that full information on the approval of the study protocol must also be provided in the manuscript.
